# Regional diversity in subsistence among early farmers in Southeast Europe revealed by archaeological organic residues

**DOI:** 10.1098/rspb.2018.2347

**Published:** 2019-01-16

**Authors:** Lucy J. E. Cramp, Jonathan Ethier, Dushka Urem-Kotsou, Clive Bonsall, Dušan Borić, Adina Boroneanţ, Richard P. Evershed, Slaviša Perić, Mélanie Roffet-Salque, Helen L. Whelton, Maria Ivanova

**Affiliations:** 1 Department of Anthropology and Archaeology, University of Bristol, 43 Woodland Road, Bristol BS8 1UU, UK; 2 Institut für Ur-und Frühgeschichte und Vorderasiatische Archäologie, Universität Heidelberg, Marstallhof 4, 69117 Heidelberg, Germany; 3 Department of History and Ethnology, Democritus University of Thrace, Tsaldari 1, Komotini 694100, Greece; 4 School of History, Classics and Archaeology, University of Edinburgh, Old Medical School, Teviot Place, Edinburgh EH8 9AG, UK; 5 The Italian Academy for Advanced Studies in America, Columbia University, 1161 Amsterdam Avenue, New York, NY 10027, USA; 6 ‘Vasile Pârvan’ Institute of Archaeology, Romanian Academy, Henri Coandă Strada 11, Bucharest 010667, Romania; 7 Organic Geochemistry Unit, School of Chemistry, University of Bristol, Cantock's Close, Bristol BS8 1TS, UK; 8 Institute of Archaeology, Knez Mihailova 35/4, 11000 Belgrade, Serbia

**Keywords:** lipid biomarkers, organic residues, neolithic, early farmer, aquatic, pottery

## Abstract

The spread of early farming across Europe from its origins in Southwest Asia was a culturally transformative process which took place over millennia. Within regions, the pace of the transition was probably related to the particular climatic and environmental conditions encountered, as well as the nature of localized hunter–gatherer and farmer interactions. The establishment of farming in the interior of the Balkans represents the first movement of Southwest Asian livestock beyond their natural climatic range, and widespread evidence now exists for early pottery being used extensively for dairying. However, pottery lipid residues from sites in the Iron Gates region of the Danube in the northern Balkans show that here, Neolithic pottery was being used predominantly for processing aquatic resources. This stands out not only within the surrounding region but also contrasts markedly with Neolithic pottery use across wider Europe. These findings provide evidence for the strategic diversity within the wider cultural and economic practices during the Neolithic, with this exceptional environmental and cultural setting offering alternative opportunities despite the dominance of farming in the wider region.

## 1. Introduction and background

Across most of Europe (with notable exceptions including the Baltic region), the transition towards an economy based upon domesticated animals and plants was accompanied by the first appearance of pottery. While the timing, pace and nature of the shift towards farming varied, the study of archaeozoological assemblages and pottery organic residues supports scenarios whereby domesticated ruminants (cattle, sheep and goat) became the predominant animals exploited, accompanied by smaller numbers of wild ruminants (e.g. cervids and aurochs) and non-ruminants (such as domesticated pig, wild boar and fish/shellfish). Alongside evidence for ruminant meat, dairy products have also been identified in pottery organic residues as early as the seventh millennium BC in Anatolia and Syria [1–4], and subsequently in Neolithic pottery from Southeastern Europe [1,5–7], the Mediterranean [8], and Central [9–11], Northwestern [12,13] and Northern [14,15] Europe. While exploitation of fish and shellfish is evidenced from Early Neolithic coastal settlements in some areas including Southeastern Europe and the Bosporus (e.g. [16,17]), this ceases to comprise the staple component of the economy, and in some regions is conspicuously absent, even leading to suggestions of an ideological taboo against aquatic foods [18].

From Southwest Asia, farming spread into Southeastern Europe, reaching the interior of the Balkans by the seventh millennium BC. This marked the point at which Southwest Asian livestock was introduced beyond their natural climatic zone into temperate Europe [6,19,20], and preferences in animal and plant species exploitation have been shown to correlate with the regional bioclimatic setting as farming dispersed northwards [19]. The study of archaeozoological assemblages and organic residues from Early Neolithic pottery from the northern Balkans and Carpathian Basin demonstrate widespread and frequent evidence for dairying; the intensification of this existing practice may have been an adaptive strategy to the new environmental conditions within which animals were being raised [6].

**Figure 1.**
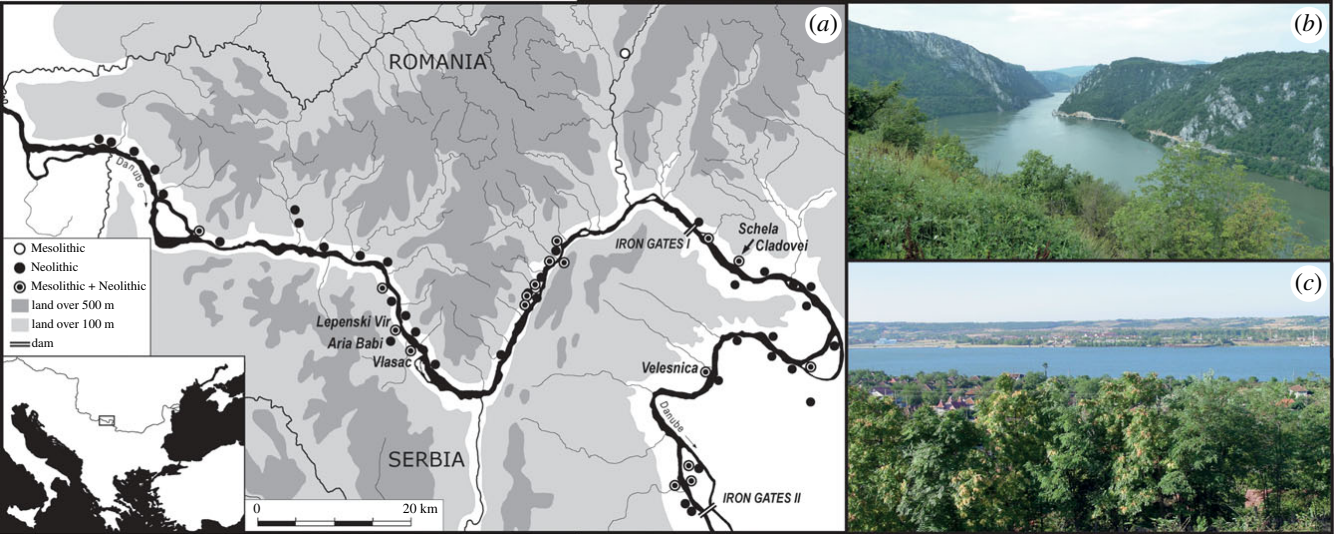
(*a*) Map of the Iron Gates region showing the location of important Mesolithic and Neolithic sites, including those investigated here. (*b,c*) The modern landscape of the gorges and downstream area, respectively.

In the northern Balkans, straddling the modern political border between Romania and Serbia, lies the Iron Gates region of the Danube, also known as the Danube Gorges (figure 1). This is a unique landscape encompassing the mountainous topography of the gorges where the Danube cuts through the junction of the Carpathian and Balkan mountain chains, and the lower lying, more open landscape of the ‘downstream area’ where the river begins its journey across the Wallachian Plain towards the Black Sea (figure 1*b,c*). This region is home to important sites that span the crucial interface between Mesolithic fisher–hunter–gatherers and the earliest farmers in the wider landscape of the central and northern Balkans. At least 18 fisher–hunter–gatherer sites dating from the Late Glacial and early Holocene were revealed by archaeological surveys and excavations undertaken during the construction of two dams between 1964 to 1971 and 1977 to 1984, some of which may have been permanent or semi-permanent settlements with semi-subterranean dwellings and formal burial grounds (e.g. [21–29]).

By the end of the seventh millennium BC, the appearance of Starčevo pottery, new crouched burial positions and domesticates in the Iron Gates region coincides with the spread of Starčevo–Körös–Criş farmers throughout the central and northern Balkans [24,30,31]. The appearance of these new cultural traits indicates the presence of, or contact with, farming communities. While faunal assemblages from early excavations in the region are affected by stratigraphic uncertainty [32], recent excavations at the downstream site of Schela Cladovei have revealed significant quantities of domesticated species during the Early Neolithic [33]. However, the gorges were arguably less well suited to farming and wild mammals continued to be important in the subsequent economy [31,34–36]. The abundance and variety of the Danube's fish resources, which included both freshwater (e.g. carp and catfish) and migratory marine (e.g. some sturgeon) species, provided a potentially rich wild resource for any groups living in the area. It seems likely therefore that farmers, who may already have incorporated some fishing into their subsistence practices, were attracted by the aquatic resources of the Danube and the fertile alluvial soils along its banks. Evidence from Sr isotopes and ancient DNA is indicative of increased mobility and genetic mixing of farmer and local forager ancestry among the populations buried in the Iron Gates [37,38] around the time Starčevo farmers are visible archaeologically in the wider landscape. Stable carbon and nitrogen isotope values of dated human bone collagen from Mesolithic and Neolithic human bones from the Iron Gates moreover indicate a broad shift towards the inclusion of more terrestrial resources in the diet, although fish continued to be a predominant component of the diets of some individuals even by the early sixth millennium BC [39–41]. That this may not have been a continuous or linear process [42,43] provides further insights into the issue of continuity of occupation and the precise nature of interactions between foragers and farmers.

Organic residues that accumulate and survive in the fabric of pottery vessels provide a means to directly determine the origins of products processed in them, such as animal fats, plant oils, leafy plants, resins, beeswax and bitumen [44] based upon their biomolecular ‘fingerprints’. Determination of the stable carbon isotope value of individual fatty acids enables further classification of animal fats, separating ruminant (e.g. cattle, sheep, goat, deer) from non-ruminant (e.g. pig, wild boar), freshwater and marine products, and fats of dairy origin [12,45,46]. The reasonably high likelihood of mixing of products in pots means that while there will be ‘over-printing’ of different biomolecular fingerprints, the stable isotope signatures of surviving animal fatty acids will be an integrated signature of potentially more than one origin and will reflect the major contributing source, or plot between categories.

Here we investigate preserved organic residues from over 200 Neolithic ceramic vessels from the Iron Gates and assess the wider implications for our understanding of Early Neolithic adaptations in Southeast Europe and the role of ceramic technology.

## 2. Material and methods

A total of 217 Neolithic sherds were selected for organic residue analysis from five settlements located in the Iron Gates region of the Lower Danube (figure 1). Two sites (Lepenski Vir and Vlasac) are located in the steep, upper gorges, positioned on narrow alluvial strips along the river bank. A third settlement, Aria Babi, is situated in the immediate hinterland of the gorges, on a hill above Lepenski Vir. The other sites, Schela Cladovei and Velesnica, are located in the more open landscape downstream of the gorges. Descriptions of individual sites, details of the sherds analysed and their archaeological contexts are provided in the electronic supplementary material (text S1, tables S1 and S2). Vessel forms sampled comprised predominantly bowls and jars, deriving from pits or ‘cultural layers’ (see electronic supplementary material, tables S1 and S2). Considerable debate continues regarding relative chronologies within and between sites. However, all pottery analysed here is of Starčevo–Criş-type, which is broadly dateable to the late seventh to early sixth millennium BC. A summary of the archaeozoological assemblages is given in figure 3 and the electronic supplementary material, table S3.

Lipid extracts were obtained and prepared from pottery sherds using well-established protocols published elsewhere [12,47] and detailed in the electronic supplementary material, text S2. Selected lipid extracts were investigated using GC/MS-SIM for high-sensitivity detection of ω-(*o*-alkylphenyl)alkanoic acids (APAAs) and dihydroxy acids (DHFAs; see electronic supplementary material, text S2 and figure S1). GC/C/IRMS analysis was performed on fatty acid methyl esters from residues identified as animal fats for the determination of *δ*^13^C values of individual fatty acids (C_16:0_ and C_18:0_). Instrumental conditions are given in the electronic supplementary material, text S2. The *δ*^13^C values were derived according to the following expression and are relative to the international standard VPDB: *δ*^13^C‰ = ((*R*_sample_ – *R*_standard_)/*R*_standard_) × 1000 where *R* = ^13^C/^12^C. The *δ*^13^C values were corrected for the carbon atoms added during methylation using a mass balance equation [48].

## 3. Results and discussion

### (a) Evidence of aquatic products in the Neolithic pottery

**Figure 2.**
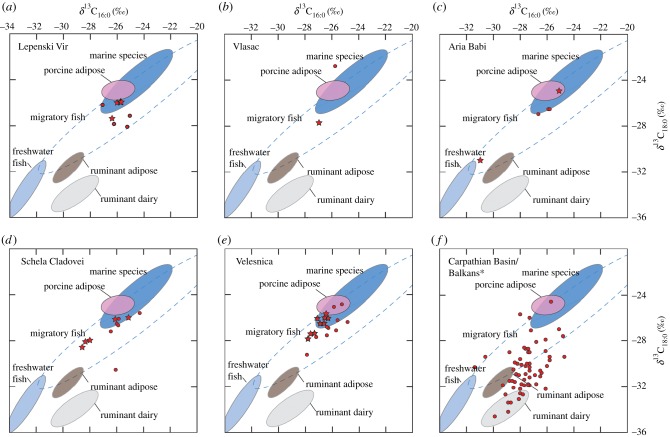
Scatter plot of *δ*^13^C_16:0_ values against *δ*^13^C_18:0_ values from Neolithic pottery residues from the Iron Gates region (*a–e*; *n* = 45) and the Carpathian Basin/northern and southern Balkans (*f*; *n* = 64; *Data from [6]). Datapoints shown as red stars indicate where APAAs and DHFAs of carbon chain length ≧ C_20_ were also observed in the residue. Coloured ellipses are 1*σ* confidence ellipses derived from modern reference datasets. Reference fats are from [12,46,50], with additional new freshwater and migratory fish data from the Severn Estuary, UK and the Irish Sea (eels and salmon). Terrestrial and freshwater modern fats have been corrected for the contribution of post-industrial carbon (+1.3‰ [51]).

**Figure 3.**
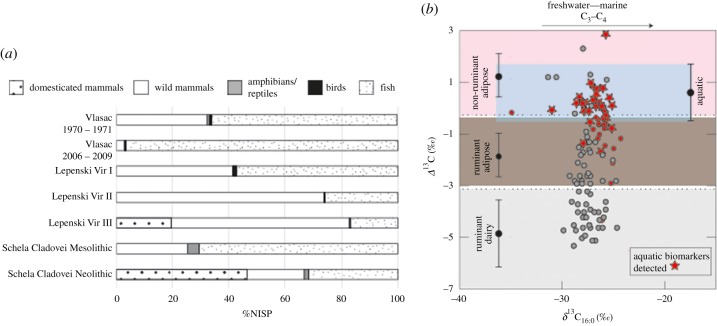
(*a*) Summary of archaeozoological assemblages from Vlasac [52,53], Lepenski Vir [34] and Schela Cladovei [33] based on NISP. Wild mammal data may include antler as this was not consistently quantified separately in the original reports. (*b*) Plot of *δ*^13^C_16:0_ against Δ^13^C (*δ*^13^C_18:0_–*δ*^13^C_16:0_) values, showing (red circles and stars) the data from the five sites investigated from the Iron Gates region (*n* = 45). By contrast, the grey circles show the stable isotope values from Neolithic pottery residues investigated from the northern and southern Balkans and Carpathian Basin, published in [6]; (*n* = 64). No aquatic biomarkers were detected in the lipid residues in pottery from the northern and southern Balkans. The ranges show the mean ± 1 s.d. from a database of reference values published in [49] (terrestrial fats); aquatic ranges as in figure 2.

From over 200 pottery sherds sampled, 45 contained preserved characterizable organic residues. The majority were dominated by saturated C_16:0_ and C_18:0_ fatty acids (see electronic supplementary material, table S2 and figure S1A–C), which are the most abundant fatty acids in degraded animal fats. The determination of the *δ*^13^C values of these individual fatty acids enables further classification of their origins [12]. While absolute *δ*^13^C values may express regional differences, plotting the Δ^13^C (*δ*^13^C_18:0_ – *δ*^13^C_16:0_) values against *δ*^13^C_16:0_ values minimizes these effects, emphasizing the differences in metabolic physiologies [49] (figures 2 and 3). In contrast to nearly all previously published Early and Middle Neolithic European investigations, the stable carbon isotope determinations show that non-ruminants comprise a major source of the products processed in the pottery from this location, with non-ruminant signatures dominant in over 50% of residues. In the Iron Gates ecosystem, and taking into account the faunal records, non-ruminants could comprise both aquatic resources from the Danube, porcine fats (wild or domestic), as well as dog. By using gas chromatography/mass spectrometry operated in selected ion monitoring mode, the widespread (70%; figure 2) presence of long-chain (≥C_20_) vicinal diols and ω-(*o*-alkylphenyl)alkanoic acids was detected in the non-ruminant residues (and in a further three residues with ruminant stable isotope signatures), which reflect the presence of precursor long-chain mono- and polyunsaturated fats respectively [46,54–58] (see electronic supplementary material, table S2 and figure S1), abundant in fresh aquatic fats. This confirms that aquatic products were regularly processed in over half of the Early Neolithic pots across all five Iron Gates settlements.

Owing to the relatively low content of C_18:0_ in aquatic fats, their contribution to the measured stable isotope signature would readily be masked isotopically by mixing with terrestrial animal fats and could plot within the ruminant ranges (see electronic supplementary material, figure S2). Therefore, aquatic resources must have been the predominant commodity contained or processed in the pots, in order for this signature to have been retained.

The stable carbon isotope values exhibit a wide range of absolute values (figure 2), which do not fall within the more isotopically depleted range usually expected for freshwater ecosystems. However, the incorporation of more enriched values can readily be explained by likely extensive processing of anadromous fish such as sturgeon, which were available in significant quantities, especially in the vicinity of whirlpools and rapids such as those near Schela Cladovei and Lepenski Vir [39,42,59]. Since most sturgeon species do not feed once they enter the freshwater river system for spawning, a marine stable carbon isotope signature would be anticipated in tissues from larger, older sturgeon, while juveniles, non-diadromous sturgeon (e.g. sterlet) and other non-migratory fish (e.g. carp) would display more isotopically depleted freshwater signatures. In between, tissues from anadromous species that continue to feed in freshwater locations such as *Huso huso* (beluga) would comprise a mixture of carbon sources, indeed, as reflected in bone collagen stable carbon isotope values of sturgeon bones from Vlasac [42]. The organic residues are an accumulation of lipids across multiple uses of the pot and so the *δ*^13^C values will provide an integrated signature of these potentially different sources of carbon.

### (b) Evidence for terrestrial animals in Neolithic pots

While cattle, sheep/goat and deer dominate in the Neolithic archaeozoological assemblages (see electronic supplementary material, table S3), fewer than half of the residues display a ruminant stable carbon isotope signature, despite the bias against the isotopic visibility of aquatic fats if products were mixed. The leanness of wild game compared with fish flesh and roe (fat comprising 1–2% of meat from modern wild deer [60,61], cf. sturgeon, catfish and carp meat in the region of 5% [62] and wild sturgeon caviar between 10 and 20% [63,64]) may have contributed to the unusually high visibility of aquatic products here, but it is evident nonetheless that at these sites, ruminants could not have been the predominant resource processed in pottery. It is also notable that dairy (and by extension, unambiguous evidence for domesticated) products are almost entirely absent, represented by only a single residue from Schela Cladovei. In stark contrast with Starčevo pottery from the wider Balkans region, it is highly unlikely that dairying comprised a significant component of the activities taking place here, although the possibility of low quantities of dairy products being masked isotopically through mixing with very high quantities of non-ruminant fat cannot be ruled out.

In addition to ruminants, non-ruminant mammals, including pig and dog are also possible components of the economy of the Iron Gates region. Pig remains have been identified among archaeozoological assemblages in low quantities from Neolithic layers [33,34] as well as the dog from both Mesolithic and Early Neolithic assemblages [34,35,52,53,65]. While the presence of aquatic biomarkers shows that the non-ruminant stable isotope signatures from residues are associated with aquatic product processing, the additional contribution of terrestrial non-ruminants to this overall signature is quite plausible and cannot be further disentangled.

### (c) The broader picture

Farming practices spread rapidly through the Balkan peninsula, probably along coastal plains and river valleys through the mountainous Balkan interior. The archaeology of the Iron Gates region is exceptional for the survival of evidence that spans this period, with abundant evidence of Late Mesolithic and Early Neolithic settlement. However, with the loss of coastal sites due to the Holocene marine transgression and relatively unexplored archaeology of the Lower Danube, it is possible that this was not an exceptional scenario in prehistory [66,67]. This surviving evidence enables closer examination of the likely diversity of Late Mesolithic and Early Neolithic practices along the Danube, the interactions that took place between peoples from different cultural backgrounds with the local environment, and their external contacts and trade networks.

**Figure 4.**
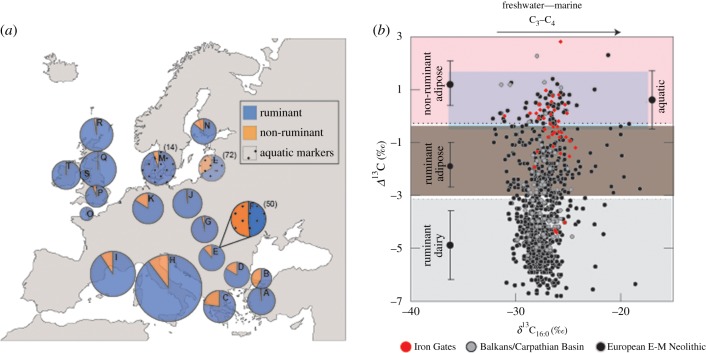
(*a*) Pie charts spanning distribution of sites from which organic residue analysis was undertaken, showing proportions of pottery lipids classified as ruminant (blue; Δ^13^C < −0.3‰) or non-ruminant (orange; Δ^13^C ≧ −0.3‰). Numbers in brackets denote the percentage of sherds containing aquatic biomarkers, where applicable. A—northwest Anatolia, Neolithic (seventh mill. BC), *n* = 65 [1]; B—Yenikapı, Neolithic (mid-seventh to late sixth mill. BC), *n* = 12 [4]; C—northern Greece, Early–Middle Neolithic (mid-seventh to mid-sixth mill. BC), *n* = 100 [5]; D—southern Balkans, Karanovo I (early sixth mill. BC), *n* = 24 [6]; E—northern Balkans, Starčevo (late seventh to early sixth mill. BC), *n* = 18 [6]; F—Iron Gates, Starčevo and Criş (early sixth mill. BC), *n* = 45 (this paper); G—Carpathian Basin, Starčevo (early sixth mill. BC), *n* = 22 [6,7]; H—Central Mediterranean, Impressa/Early Neolithic (sixth to fifth mill. BC), *n* = 29 [8]; I—Western Mediterranean, Impressa/Cardial (sixth mill. BC), *n* = 43 [8]; J—Poland, LBK (late sixth to early fifth mill. BC), *n* = 67 [9]; K—Germany, LBK (mid- to late sixth mill. BC), *n* = 25 [10]; L—Lithuania, Globular Amphora Culture and Rzucewo (late fourth to mid-third mill. BC), *n* = 18 [70]; M—southern Scandinavia, TRB (fourth mill. BC), *n* = 56 [15]; N—southern Finland, Corded Ware (third mill. BC), *n* = 7 [14]; O—Channel Isles, Cerny/Early Castellic, Le Pinacle/VSG (fifth mill. BC), *n* = 8 [13]; P—southern Britain, Early Neolithic (fourth mill. BC), *n* = 165 [12]; Q—northern Britain, Carinated Bowl/Unstan/Early Grooved Ware (late fifth to fourth mill. BC), *n* = 73 [13]; R—Northern and Western Isles of Scotland, Hebridean/Unstan/Early Grooved Ware (mid–late fourth mill. BC), *n* = 43 [13]; S—Isle of Man, Ronaldsway (late fifth to mid-fourth mill. BC), *n* = 10 [13]; T—Island of Ireland, Carinated Bowl (late fifth–fourth mill. BC), *n* = 106 [68]. Plot (*b*) Scatter plot of compound-specific isotope values of *δ*^13^C_16:0_ values plotted against Δ^13^C (*δ*^13^C_18:0_–*δ*^13^C_16:0_), showing data from this paper (red), the Balkans and Carpathian Basin [6] (grey) and Early–Middle Neolithic pottery from Europe (black; all other references above, except [4] not plotted). Ranges shown are as for figure 3. Total no. data points included = 904.

The pottery residues, which date to the first half of the sixth millennium BC, demonstrate that Starčevo–Körös–Criş traditions of Early Neolithic pottery were being used for diverse purposes within the wider Balkan region. Along the banks of the Danube in the Iron Gates, pots are strongly associated with the processing of abundant riverine resources. By contrast, some 100 km away further north in the Balkans and on the Great Hungarian Plain, broadly contemporaneous Starčevo pots were being used predominantly for terrestrial resources, even at riverine locations [6,7], and in particular for processing dairy products. This aligns with local archaeozoological assemblages dominated by domesticated cattle, sheep/goat and deer. On a wider scale, organic residues from Early Neolithic pottery from Southeastern Europe, the Southern and Central Mediterranean and Northwest Europe display an emphasis upon terrestrial, ruminant-based commodities, including varying degrees of dairy products, even at coastal or lacustrine settlements (e.g. [1–15,68,69]; figure 4) and where there is otherwise evidence for fishing and shellfish exploitation, such as Northwest Anatolia [71].

Within the Iron Gates region, the presence of a wider range and higher abundance of fish remains in the Neolithic is now becoming recognized from more recent excavations and re-analyses of older material [33,39,42,72]. In addition to carp and catfish, the exploitation of sturgeon was an important and valued source of protein for prehistoric communities inhabiting the Iron Gates Mesolithic (e.g. [32,33]), and bones have also been identified among Neolithic faunal assemblages at Schela Cladovei [33,59]. Although this was initially thought to be unusual [24,33], re-analysis of faunal remains from contexts dated to 6300–5500 BC from Lepenski Vir has also identified Acipenseridae bones further upstream [72]. It is possible that misidentification and taphonomic bias against larger (i.e. older) sturgeon due to the re-absorption of bone minerals may both have led to under-estimation of the contribution of sturgeon [32,42,59,72]. The exceptional size of sturgeon (reaching several hundred kilograms), observed frequency and likely under-representation of their remains indicate that this and other aquatic species were probably an important source of food. It is possible that sturgeon capture was undertaken on a seasonal basis, as practised in more recent times [32,59], because sturgeon tend to swim upriver for spawning in spring/summer [73], but even if so, they may have been dried, smoked or otherwise preserved for year-round consumption.

The C and N stable isotope values obtained from human bone collagen from dated individuals broadly indicate that some humans were consuming a diet with an increased terrestrial component around the time of the appearance of pottery and livestock in the Iron Gates. The evidence nonetheless indicates a continued role for aquatic resources [39,40,42,43,74–77] and a higher degree of fish consumption compared with Early Neolithic sites further from the Danube or other large rivers such as the Tisza [30,78]. While the users of pottery in the Iron Gates may have had already some familiarity with fishing practices, the high visibility of aquatic organic residues is very unusual in farmer-type pottery and more closely akin to organic residues in pottery used by hunter–fisher–foragers around the Baltic Sea in the fifth to fourth millennia BC [15,16,70]. At these locations, between 20 and 70% of residues were attributed to a non-ruminant origin based on stable isotope compositions, and between 20 and 70% of total residues contained evidence for aquatic biomarkers. In these same regions, pottery types that have stronger affinities with early farming cultures contain residues that are also indicative of continued, but usually less-visible, aquatic resource processing. This is witnessed to some extent in Neolithic TRB pottery from the western Baltic [15], and to a greater extent from residues recovered from Globular Amphora Ware and Rzucewo pottery from Lithuania [70]. In these regions, the rich resources of coastal and lacustrine habitats and environments that were challenging for farming may have combined to result in a significant degree of wild resource exploitation alongside the presence of domesticates.

In the Iron Gates, the topography of the gorge would have placed restrictions upon farming. Localized pockets of fertile land suitable for cultivation or pasture would have been available, although the more open and moderate relief of the lower Danube, where the river is flanked by fertile alluvial terraces of Pleistocene and Holocene age, would have offered better opportunities for farming. Despite terrestrial animal fats being universally widespread in prehistoric pottery, the pottery organic residues here do not show any greater indication of animal husbandry as a significant practice even in this downstream area. Among possible explanations for this pattern are that these pottery users had diets still drawing heavily on aquatic resources and only limited ruminant and dairy products, or pottery was being used here for a restricted purpose, with other resources prepared differently (e.g. spit roasting). A third possibility is that if the processing of a resource was frequently done close to the point of procurement, then this pattern may reflect the proximity of sampled contexts to the Danube banks which were not necessarily places where livestock was kept. A final possibility is that these sites were seasonally occupied, as may have been the case for some Neolithic sites in the Upper Gorge [24]. Despite the importance of fishing in the Iron Gates region over preceding millennia, the organic residues in pottery can be seen to represent a change in how fish was processed, with pots now being used for preparation, a technology that may have facilitated new activities such as making stews and soups or oil rendering.

To date, multiple scenarios of cultural interaction between foragers and farmers, as well as localized social transformations, have been proposed (e.g. [25,26,32,39,72,79–82]). While at sites such as Schela Cladovei, there is a hiatus between the Late Mesolithic occupation and re-occupation by groups displaying characteristically Neolithic traits [24], recent studies of both ancient DNA and strontium isotope signatures from human tooth enamel now provide evidence for the interaction of immigrant farmers with local foraging communities, including burial of non-locals in a ‘Mesolithic’ tradition [37,38,83,84], leading some authors to recognize a ‘transformational’ phase between the Late Mesolithic and Early Neolithic [26,72]. Our research has demonstrated the continued preparation of riverine resources in the Iron Gates, now using Neolithic pottery, which may relate to both the resource base encountered and cultural interactions occurring within pre-existing and newly formed social networks.

## 4. Conclusion

The Early Neolithic pottery residues from the Iron Gates region confirm interpretations from archaeozoological and stable isotope evidence that the use of aquatic resources continued into the early sixth millennium BC, echoing Late Mesolithic traditions, despite farming being practised in the wider region. The widespread use of Starčevo–Criş pottery in the Iron Gates for processing riverine resources is in marked contrast to its role for preparing ruminant meat and dairy products across the northern Balkans, Carpathian Basin, and indeed most Early and Middle Neolithic pottery from across wider Europe, even where fishing was probably practised. This suggests that the pottery was being used for a relatively specialized purpose at this location, whether it was being brought by farmers making use of the localized resources, incorporated into existing practices in the Iron Gates, or a combination of the two. Either way, it represents a new method for processing fish that was introduced within the Iron Gates and demonstrates a clear diversity in the function of this type of pottery within different cultural and ecological contexts.

## Supplementary Material

Supplementary information to Cramp et al. Regional diversity in subsistence among early farmers in Southeast Europe revealed by archaeological organic residues.

## Data accessibility

The datasets supporting this article have been uploaded as part of the electronic supplementary material.
