# Supplementary material for: Regional diversity in subsistence among early farmers in Southeast Europe revealed by archaeological organic residues
Source: Proc Biol Sci. 2019 Jan 16;286(1894):20182347. doi: 10.1098/rspb.2018.2347 (PMC6367183; doi:10.1098/rspb.2018.2347)
Supplement: Supplementary information to Cramp et al. Regional diversity in subsistence among early farmers in Southeast Europe revealed by archaeological organic residues. [file rspb20182347supp1.pdf]

Supplementary materials

Cramp, LJE, Ethier, J, Urem-Kotsou, D, Bonsall, B, Borić, D, Boroneanț, A, Evershed, RP, Perić, S, Roffet-Salque, M,Whelton, H and Ivanova, I. *Regional diversity in subsistence among early farmers in Southeast Europe revealed by archaeological pottery organic residues.*

Text S1. Site descriptions
Text S2. Analytical Procedures

Table S1. Contextual information for pottery discussed
Table S2. Summary of lipids characterised from the five settlements
Table S3. Summary of published faunal information from Vlasac, Lepenski Vir and Schela Cladovei

Figure S1. Partial gas chromatograms showing typical fatty acid distributions
Figure S2. Plot of $\delta^{13}C_{16:0}$ against $\Delta^{13}C$ ($\delta^{13}C_{18:0}$ – $\delta^{13}C_{16:0}$) mean and full ranges from analyses of reference species

Text S1. Site descriptions

Vlasac

Vlasac was discovered in 1970 and partially excavated during the final stages of construction of the Iron Gates I dam by Srejović and Letica [1]. Over 100 Mesolithic burials were discovered, along with dwelling features and stone-lined hearths. Neolithic pottery was also found in the upper levels of the site. Initial dating led to interpretations of a period of abandonment between the Late Mesolithic and Neolithic occupation at around 6400 cal BC [2]. The site was reinvestigated in 2006–9 after reports of bones being washed out from the eroding riverbank. Previously unidentified areas were excavated, with new stratigraphic and radiocarbon evidence suggesting that at least part of the site had been used between 6300 – 5900 cal BC [3,4]. Archaeozoological studies suggest wild resources were a primary aspect of the Mesolithic economy, including red deer, wild boar, carp and sturgeon [4,5]. Dog was also numerous.

The only date relating to a pottery-containing context at Vlasac is an AMS ^{14}C determination of 6006 – 5838 cal BC (95 % confidence) on a red deer antler stratified below a layer with pottery [4]. However, this is only a temporary guideline for the overall timing of the introduction of pottery at Vlasac. Thirty-nine Starčevo-type potsherds from the 2006 excavation season were analyzed for organic residues (Table S1A). Two sherds yielded sufficiently high concentrations of lipid for further analysis. Both derived from general Starčevo 'cultural layers', one of which lies stratigraphically above the dated deer skull, and the other from the southern edge of the same trench, which bears no direct stratigraphic relationship to the skull.

Lepenski Vir

The site of Lepenski Vir lies in the upper gorge on the right bank of the Danube in present-day Serbia. In excavations from 1965 to 1970, D. Srejović uncovered what he interpreted as an uninterrupted sequence of Mesolithic and Early Neolithic occupations comprising four main phases – Proto-Lepenski Vir (Early Mesolithic), Lepenski Vir I and II (Late Mesolithic) and Lepenski Vir III (Early Neolithic) [6]. The LVI-II occupation phases were associated with trapezoidal structures or 'houses' with lime plaster floors, extended inhumations and carved stone boulders. Lepenski Vir III was characterized by material

culture characteristic of the Early Neolithic of the central Balkans with pits, ovens, bone tools, polished stone axes, ‘Balkan flint’, crouched burials, and Starčevo-style pottery.

The relative and absolute chronology of Lepenski Vir has been much debated, and conflicting chronologies have been proposed (e.g. [7-10]. Radiocarbon dates on bulk charcoal and subsequent AMS ^{14}C dating of human and animal bones from Srejović’s excavations [9, 11-15] have revealed significant gaps in the sequence, but apparently uninterrupted occupation between *c*. 6200–5800 cal BC, spanning the Mesolithic–Neolithic interface in the wider region.

The first Neolithic-style (crouched) burials at Lepenski Vir and the earliest direct dates on bones from domestic livestock fall in the later part of this time-range, between 6000-5800 cal BC, and evidently postdate the period of the trapezoidal buildings, though wild game (red deer, wild boar, aurochs and fish) still dominate the LV III faunal assemblage [16].

There are no radiocarbon dates from secure contexts with Starčevo pottery. While some chronological models would see pottery as early as 6300 cal BC (e.g. [9, 10], others date its appearance *c*. 6000 BC [17]. The presence of a few Starčevo sherds inside some trapezoidal structures of LVI-II was interpreted by Srejović as intrusive, but this has not been accepted by some scholars (e.g. [7, 18-9] who view it as evidence of the Mesolithic inhabitants interacting with farmers – the beginning of the Neolithic transition.

All 41 sherds from Lepenski Vir sampled here derive from contexts attributed to Lepenski Vir III (Table S1 B). These were sampled from a range of vessel shapes (plates, bowls and jars, in both fine and coarse wares). Eight extracts with sufficient concentrations of lipids were subjected to further biomolecular and isotopic analysis.

Aria Babi

Lying above Lepenski Vir on the Košo Hill, the recently discovered site of Aria Babi was excavated in 2004. This was part of a wider investigation into the prehistory of northeast Serbia led by D. Borić and M. Jevtić, which involved systematic survey of the hinterland on the Serbian side of the Danube [20-22]. This is believed to be a single occupation site, and whilst no dwellings were discovered, pits containing Early Neolithic (Starčevo) pottery and stone artefacts were found. There are no radiocarbon dates due to a lack of organic remains; however, in terms of vessel shapes and decorative techniques the pottery from Aria Babi has been compared to that from Lepenski Vir III [20]. Since no animal or human remains were

preserved, organic residue analysis of pottery is the only means of gaining dietary information from this site. Twenty-one pot sherds from the 2004 excavation were analysed, deriving from Test Trench 6 and Spit 1 of Trench 1 (Table S1 C). From these, five were taken forward for further biomolecular and stable isotope analysis.

Schela Cladovei

Schela Cladovei is situated in the downstream area of the Iron Gates in a suburb of Drobeta Turnu-Severin on the left bank of the Danube in Romania. The site lies on an alluvial flat bordering the Danube. It had been extensively excavated by V. Boroneanţ between 1965 and 1991. Since 1992 it has been a Romanian-British research project, initially (1992-6) co-directed by V. Boroneanţ and C. Bonsall, and latterly (2007 onwards) by A. Boroneanţ and C. Bonsall [23]. Unlike Lepenski Vir, the habitation sequence of Schela Cladovei shows an interruption, with a Late Mesolithic occupation between c.7200 and 6300 cal BC followed by a hiatus of several centuries. Schela Cladovei was reoccupied c. 6000 cal BC by a group whose material culture displays characteristic Early Neolithic (Starčevo-Criş culture) traits [8, 24]. In the faunal assemblage, domesticates (sheep/goat, cattle and pig) outnumber wild mammals, but fish remains are numerous [25].

In total, 49 ceramic sherds from Schela Cladovei were sampled and lipid extracted, originating from Early Neolithic pits in the current excavations of Area VII (Table S1 D). The sherds were selected from a range of vessel shapes (plates, bowls and jars) in both medium and coarse wares. There are no radiocarbon dates as yet from Area VII, but on the basis of similarities with the ceramic repertoire of nearby Area VI (excavated 1992-6), the pottery likely dates to 6000–5700 cal BC. Twelve lipid extracts with sufficient concentrations of lipids were prepared for further biomolecular and isotopic analysis.

Velesnica

Velesnica is a multi-phase open-air settlement situated on a small terrace on the right bank of the Danube, which was first excavated systematically by R. Vasić between 1980 and 1984 after a number of small-scale excavations from the beginning of the 20th century. A Starčevo cultural layer over a metre thick was recorded, within which two occupation horizons were distinguished [26]. Animal bones lying below the Starčevo layer may relate to earlier Mesolithic occupation, and comprise a range of wild species including red deer, aurochs, and wild boar [26: 227]. Fauna assigned to the earlier Starčevo horizon are

dominated by wild species (>95 %; [26: 238] but may be a mixture of material from Mesolithic and Neolithic occupations. The Early Neolithic finds included the remains of at least one rectangular building, hearths, pottery, stone and bone tools, possible stone 'altars' and three graves containing up to nine individuals in mainly crouched body positions [26]. In total, 67 pottery sherds were sampled from Velesnica, all deriving from the earlier Starčevo horizon (Table S1 E). Vessel forms included a range of shallow and deeper bowls and jars in both fine and coarse wares, frequently decorated with impressions, engravings or applied and decorated horizontal bands. With one exception, radiocarbon dates on bone collagen from Burial 2, believed by the excavator to relate to the earlier Starčevo horizon [26], cluster between c. 6100–6000 cal BC after correction for freshwater reservoir effect [27]. These dates for crouched burials are somewhat earlier than those from Lepenski Vir, which may indicate that the Neolithic transition occurred slightly earlier in the downstream area of the Iron Gates. Of the 67 sherds sampled, 19 contained sufficient residues for further biomolecular and isotopic analysis.

1. Srejović D, Letica Z. 1978 *Vlasac: Mezolitsko naselje u Djerdapu (I arheologija)* (*Vlasac. A Mesolithic settlement in the Iron Gates I*). Belgrade, Srpska akademija nauka I umetnosti.
2. Bonsall C, Macklin M, Payton R, Boroneant V. 2002 Climate, floods and river gods: environmental change and the Meso-Neolithic transitions in southeast Europe. *Before Farming* **2002 (3-4),** 1 – 15.
3. Borić D. 2006 New Discoveries at the Mesolithic-Early Neolithic Site of Vlasac: Preliminary Notes. *Mesolithic Miscellany* **18(1)**, 7 – 14.
4. Borić D, French C, Stefanović S, Dimitrijević V, Cristiani E, Gurova M, Antonović D, Allué E, Filipović D. 2014 Late Mesolithic lifeways and deathways at Vlasac (Serbia). *Journal of Field Archaeology* **39(1)**, 4 – 31.
5. Bökönyi S. 1978 The vertebrate fauna of Vlasac. In *Vlasac: Mezolitsko naselje u Djerdapu, vol. 2* (ed M Garašanin), pp. 35 – 65. Belgrade, Srpska akademija nauka i umetnosti.
6. Srejović D. 1971 Die Lepenski Vir-Kultur und der Beginn der Jungsteinzeit an der mittleren Donau. In *Die Anfänge des Neolithikums vom Orient bis Nordeuropa, Teil 2: Östliches Mitteleuropa*, *Fundamenta* (ed H Schwabedissen), pp. 1 – 19. Köln/Wien, Monographien zur Urgeschichte, Reihe A, Band 3.
7. Radovanović I. 1996 *The Iron Gates Mesolithic*. Ann Arbor, MI, International Monographs in Prehistory (Archaeological Series 11).

8. Bonsall C. 2008 The Mesolithic of the Iron Gates. In *Mesolithic Europe* (eds G Bailey, P Spikins), pp. 238 – 278. Cambridge, Cambridge University Press.
9. Borić D, Dimitrijević V. 2009 Absolute chronology and stratigraphy of Lepenski Vir. *Starinar* **57**, 9 – 55.
10. Perić S, Nikolić, D. 2016 *Lepenski Vir: Stratigraphy, Chronology and Periodisation: (Excavations 1966).* Beograd Arheološki institute.
11. Quitta H. 1975 Die Radiocarbondaten und ihre historische Interpretation. In *Lepenski Vir: eine vorgeschichtliche Geburtsstätte europäischer Kultur* (ed D Srejović), pp. 272 – 85. Bergisch Gladbach.
12. Whittle A, Bartosiewicz L, Borić D, Pettitt P, Richards M. 2002 In the beginning: new radiocarbon dates for the early Neolithic in northern Serbia and south-east Hungary. *Antaeus* **25**, 63 – 117.
13. Bonsall C *et al*. 1997 Mesolithic and early Neolithic in the Iron Gates: a palaeodietary perspective. *J Euro Archaeol* **5(1)**, 50 – 92.
14. Bonsall C *et al*. 2000 Stable isotopes, radiocarbon and the Mesolithic-Neolithic transition in the Iron Gates. *Documenta Praehistorica* **27**, 119 – 132.
15. Borić *et al*. 2018 High-resolution AMS dating of architecture, boulder artworks and the transition to farming at Lepenski Vir. Scientific Reports 8: 14221 https://doi.org/10.1038/s41598-018-31884-7
16. Bökönyi S. 1970 Animal remains from Lepenski Vir. *Science* **167(3926)**, 1702 – 4.
17. Bonsall C, Radovanović I, Roksandic M, Cook G, Higham T, Pickard C. 2008 Dating burial practices and architecture at Lepenski Vir. *The Iron Gates in prehistory: new perspectives, BAR International Series 1893* (eds C Bonsall, V Boroneanţ, I Radovanović), pp. 39 – 54. Oxford, Archaeopress.
18. Borić, D. 2002 The Lepenski Vir conundrum. Reinterpretation of the Mesolithic and Neolithic and sequences in the Danube Gorges. *Antiquity* **76**, 1026 – 1039.
19. Voytek B, Tringham R. 1989 Rethinking the Mesolithic: the case of South-east Europe. In *The Mesolithic in Europe. Papers Presented at the Third International Symposium, Edinburgh 1985* (ed C Bonsall), pp. 492 – 500. Edinburgh, John Donald.
20. Borić D. 2007 Mesolithic–Neolithic interactions in the Danube Gorges. In *Mesolithic/Neolithic interactions in the Balkans and in the Middle Danube basin. BAR International Series 1727* (eds J Kozłowski, M Nowak), pp. 31 – 45. Oxford, Archaeopress.

21. Borić D. 2011 Adaptations and transformations of the Danube Gorges foragers (c. 13,000–5500 cal BC): An overview. In *Beginnings – New Research in the Appearance of the Neolithic between Northwest Anatolia and the Carpathian Basin* (ed R Krauß), pp. 157 – 203. Rahden/Westf Verlag, Marie Leidorf GmbH.
22. Antonović D, Dimić V, Starović A, Borić D. 2017 Ground Stone Artefacts from Aria Babi. In *From Hunter-Gatherers to Farmers: Human adaptations at the end of the Pleistocene and the first part of the Holocene. Papers in Honour of Clive Bonsall* (eds M Mărgărit, A Boroneanț), pp. 135 – 147. Editura Cetate de Scaun,Târgovişte.
23. Boroneanț A, Bonsall C. 2013 1965–1968 excavations at Schela Cladovei (Romania) revisited. In *Unconformist Archaeology. Papers in Honour of Paolo Biag*i. *BAR International Series 2528* (ed Starnini E), pp. 35 – 54. Oxford, Archaeopress.
24. Bonsall C. 2007 When was the Neolithic transition in the Iron Gates? In *A short walk through the Balkans: the first Farmers of the Carpathian Basin and adjacent regions* (eds M Spataro M, P Biagi), pp. 53 – 66. Trieste, Quaderno 12, Società Preistoria Protostoria Friuli-V.G.
25. Bartosiewicz L, Boroneanţ V, Bonsall C, Stallibrass S. 2001 New data on the prehistoric fauna of the Iron Gates: a case study from Schela Cladovei, Romania. In *From the Mesolithic to the Neolithic: proceedings of the International Archaeological conference held in the Damjanich Museum of Szolnok, September 22–27, 1996* (eds R Kertész, J Makkay), pp. 15 – 21. Budapest, Archaeolingua.
26. Vasić R. 2008 Velesnica and the Lepenski Vir culture. In *The Iron Gates in prehistory: new perspectives* (eds C Bonsall C, V Boroneanţ, I Radovanović), pp. 39 – 54I. Oxford, BAR International Series 1893.
27. Bonsall C, Vasić R, Boroneanț A, Roksandic M, Soficaru A, McSweeney K, Evatt A, Agaraiuja U, Pickard C, Dimitrijević V, Higham T, Hamilton D, Cook G. 2015 New AMS dates for human remains from Stone Age sites in the Iron Gates reach of the Danube, Southeast Europe. *Radiocarbon* **57(1),** 3 – 4.

Text S2. Analytical Procedures

The chemical extraction procedure followed one of two well-established protocols [1,2] depending on the timeframe of analysis. Rigorous testing has indicated that the later, acidified methanol extraction procedure leads to indistinguishable isotope measurements but a higher lipid yield [2].

I Pottery from Velesnica, Serbia

Solvent and base extraction: Portions of the pottery were cleaned using a modelling drill to remove the outer surfaces. Approximately 2 g was then removed using a chisel, prior to crushing to powder using a mortar and pestle. After the addition of 20 µg *n*-tetratriacontane as a quantitative internal standard, lipids were extracted using 2 x 10 mL $CHCl_3$/MeOH (2:1 *v/v*) via sonication. The supernatant was centrifuged and concentrated to approximately 1 mL under a gentle stream of N_2. Aliquots were filtered through a silica column prior to derivatization.

Base extractions were performed upon selected previously solvent-extracted sherd fabric. This was in order to release lipids that remained bound to the ceramic fabric after solvent extraction. Five mL 0.5M NaOH/MeOH was added to 1 g ceramic fabric and the mixture heated (70 °C, 1h). After cooling to room temperature, the mixture was acidified to pH 3 using 1M aqueous HCl, and the lipids extracted using 3 x 3 mL $CHCl_3$ [3].

GC-FID and GC/MS of trimethylsilyl (TMS) derivatives: Aliquots of the solvent and alkaline extracts were derivatized using 40 µL *N,O*-bis(trimethylsilyl)trifluoroacetamide (BSTFA) containing 1% trimethylchlorosilane (TMCS; Sigma Aldrich). Derivatized lipid extracts were dissolved in an appropriate volume of hexane for screening and quantification by high temperature gas chromatography (HTGC-FID), using an Agilent 7890 fitted with a fused silica capillary column with a 100% dimethylpolysiloxane stationary phase (Agilent J & W, DB1-HT; 15 m x 0.32 mm i.d., 0.1 µm film thickness). The oven temperature programme comprised an isothermal at 50°C for 2 min, ramping to 350 °C at 10 °C min^{-1}, followed by a 15 min isothermal. Alkaline extracts were immediately introduced via a PTV injector set to splitless mode onto a GC/MS fitted with a fused silica capillary column with a 100% dimethyl polysiloxane stationary phase (Agilent J&W, CP-Sil 5 CB, 60 m x 0.25 mm i.d., 0.1 µm film thickness). The instrument was a ThermoFinnigan single quadrupole TraceMS run in EI mode (electron energy 70 eV, scan time of 0.6 s). Samples were first run in full scan mode (*m/z* 50–650) and then SIM, scanning for the two cleavage fragments and [M-15] ions for the most common positional isomers of tris-TMS C_{18}–C_{22} DHFAs (*m/z* 215, 243, 317, 345, 517, 545 and 573). The temperature programme comprised an isothermal hold at 70 °C for 2 min,

ramping to 220 °C at 10 °C min^{-1}, followed by the second ramp at 4°C min^{-1} to 300 °C, with a 10 min isothermal hold [4].

GC/MS and GC/MS-SIM of fatty acid methyl esters (FAMEs): After screening, aliquots of selected TLEs were hydrolysed (2 mL 0.5 M NaOH/MeOH, 70°C 1h) and after the extraction of the neutral fraction using 3 x 3 mL hexane, acidified to pH3 and the free fatty acids extracted (3 x 3 mL $CHCl_3$) and methylated using 100 µL BF_3/MeOH (75°C, 1 h). The fatty acid methyl esters (FAMEs) were screened using an Agilent 5890 GC fitted with a fused silica capillary column with a high cyano-modified cyanopropyl polysilphenylenesiloxane stationary phase (Agilent J & W, VF-23ms, 60 m x 0.32 mm i.d., 0.15 µm film thickness). They were then analysed using a ThermoFinnigan single quadrupole TraceMS run in EI mode (electron energy 70 eV) fitted with the same polar column and an oven temperature programme that comprised an isothermal at 70°C, ramping to 100 °C at 10 °C min^{-1} and a second ramp to 250 °C at 4 °C min^{-1}. The GC/MS was operated in both full scan (*m/z* 50 – 650) and selected ion monitoring (*m/z* 105, 262, 290, 318 and 346) mode [4]. Data were collected and analysed using Xcalibur software (v. 2.0) and a NIST spectral database.

GC-C-IRMS of FAMEs: GC-C-IRMS analyses were performed using an Agilent 6890 GC coupled to a $Delta^{plus}$ XL via a Finnegan MAT GC combustion III interface. The GC was fitted with a fused silica capillary column with a 100% dimethylpolysiloxane stationary phase (Agilent J & W CP-Sil 5 CB, 50 m x 0.32 i.d. 0.12 µm film thickness). Samples were introduced via a split/splitless injector in splitless mode. The temperature programme comprised a 2 min isothermal at 50°C followed by an increase of 10 °C min^{-1} to 300 °C and a 10 min isothermal. The injector temperature started at 70 °C and increased to 300 °C at a rate of 600 °C min^{-1} and was then held for 30 min. Cu, NI, Pt wires (0.1 mm) were used in the alumina combustion reactor (0.5 mm i.d.). The combustion reactor temperature was maintained at 950 °C. Faraday cups were used to detect ions of *m/z* 44 ($^{12}C^{16}O_2$), 45 ($^{13}C^{16}O_2$ and $^{12}C^{17}O^{16}O$) and 46 ($^{12}C^{18}O^{16}O$). Results were calibrated against reference CO_2 which was injected directly into the source 3 x at the beginning and end of the run. All samples were run in duplicate and any runs of dubious integrity discarded and repeated. External standards were run every 4 runs. $\delta^{13}C$ values required correction to take into account the extra carbon atom added during methylation using a mass balance equation [5].

II Pottery from Aria Babi, Vlasac, Lepenski Vir, Schela Cladovei

Lipid extraction

Portions of the pottery were cleaned using a modelling drill to remove the outer surfaces. Approximately 2 g was then removed using a chisel, prior to crushing to powder using a mortar and pestle. After the addition of 20 μg *n*-tetratriacontane as a quantitative internal standard, lipids were simultaneously extracted and transesterified to fatty acid methyl esters using 5 mL of 2% *v/v* H_2SO_4/MeOH (70 °C, 1 h). Once cooled, the supernatant was transferred and centrifuged and the supernatant transferred again to a clean tube (tube II) and 2 mL double-distilled DCM-extracted water added. To ensure recovery of lipid not fully solubilized by the methanol solution, 2 mL hexane was added to the original potsherd in tube I, mixed and the supernatant added to tube II; the aqueous MeOH and hexane layer were allowed to separate, and the hexane layer transferred into a final vial. This step was repeated twice, before adding 2 x 2 mL hexane to the aqueous layer in tube II only. All extracts were combined before blowing down under nitrogen at 40 °C. Aliquots of the lipid extracts were derivatised using BSTFA containing 1% TMCS (BSTFA, 70°C, 1 h) to derivatise any hydroxylated compounds to TMS ethers. Excess BSTFA was removed under N_2 and the extract dissolved in hexane.

GC-FID and GC/MS of FAMEs and TMS derivatives:

Derivatised FAMEs were screened and quantified using gas chromatography with reference to the known quantity of internal standard. Conditions for (i) for Aria Babi and Vlasac involved an Agilent 7890a GC-FID fitted with a fused silica capillary column with a 100% dimethyl polysiloxane stationary phase (Agilent J & W, DB1-HT, 15 m x 0.32 mm i.d., 0.1 μm film thickness) using He as carrier gas. The oven temperature programme consisted of an isothermal at 50 °C for 2 min, ramping to 350 °C at 10°C min^{-1}, followed by a 15 min isothermal at 350 °C (ii) for Lepenski Vir and Schela Cladovei, the instruments used were an Agilent 7890a or an Agilent 7820 GC-FID. The Agilent 7890a was fitted with a fused silica capillary column with a 100% dimethyl polysiloxane stationary phase (Agilent J & W, DB1-HT, 15 m x 0.32 mm i.d., 0.1 μm film thickness) using He as carrier gas. The oven temperature programme consisted of an isothermal at 50 °C for 2 min, ramping to 350 °C at 10 °C min^{-1}, followed by a 10 min isothermal at 350 °C. The Agilent 7820 was fitted with a fused silica capillary column with a 100% dimethylpolysiloxane stationary phase (Agilent J & W, DB1-HT, 15 m x 0.32 mm i.d., 0.1 μm film thickness) using He as carrier gas. The oven temperature programme consisted of an isothermal at 50 °C for 2 min, ramping to 300 °C at 10 °C min^{-1}, followed by a 10 min isothermal at 300 °C. Data were analysed using Agilent Chemstation software. If no hydroxylated compounds were identified (as their TMS derivatives), further analysis was performed without derivatisation.

GC/MS analyses were performed using a ThermoFinnegan single quadrupole TraceMS run in EI mode (electron energy 70 eV) with a PTV injector in splitless mode fitted with a fused silica capillary column with a high cyano-modified cyanopropyl polysilphenylenesiloxane stationary phase (Agilent J & W, VF-23ms, 60 m x 0.32 mm i.d., 0.15 µm film thickness). The oven temperature programme consisted of an isothermal at 50 °C for 2 min, ramping to 100 °C at 10°C min^{-1} followed by a second ramp to 240 °C at 4°C $min^{-1}$and an isothermal at 240 °C for 15 min. The GC/MS was operated in both full scan (*m/z* 50 – 650) and selected ion monitoring (*m/z* 105, 262, 290, 318 and 346) mode with a scan time of 0.6 sec^{-1} to detect the presence of C_{16} – C_{22} APAAs at high sensitivity [4].

Additional aliquots were derivatised using BSTFA and analysed using GC/MS and GC/MS-SIM to identify the presence of dihydroxy acids as bis-TMS methyl esters. These analyses were performed using a ThermoScientific Trace 1300 gas chromatograph coupled to an ISQ single quadrupole mass spectrometer, operated in electron ionisation (EI) mode at 70 eV. Samples were introduced *via* a PTV injector set to splitless mode onto a 50 m × 0.32 mm fused silica capillary column coated with a 100% dimethylpolysiloxane stationary phase (Restek, Rtx-1, 0.17 µm film thickness). The GC temperature programme for was set to hold at 50 °C for 1 min, followed by a gradient increase to 300 °C at 10 °C min^{-1}, and then held isothermally at 300°C for 8 min. Helium was used as the carrier gas, set to a constant flow of 2 mL min^{-1}. The MS was set to operate in selected ion monitoring (SIM) mode, acquiring at *m/z* 159, 187, 215, 231, 259, 287, 431, 443, ,459, 471, 487, 499, 515 at 0.757 scans s^{-1}. Data were collected and analysed using Xcalibur software (v. 3.0) and a NIST spectral database [4].

Gas Chromatography-Combustion-Isotope Ratio Mass Spectrometry for $\delta^{13}C$ Analysis (GC-C-IRMS): GC-C-IRMS analyses were performed using an Agilent 7890A GC coupled to an Isoprime 100 MS via an Isoprime GC5 combustion interface. The GC was fitted with a fused silica capillary column with a non-polar 100% dimethylpolysiloxane stationary phase (Agilent J & W, HP-1, 50 m x 0.32 mm i.d., 0.17 µm film thickness). Samples were introduced via a split/splitless injector in splitless mode at 300 °C. The temperature programme consisted of a 2 min isothermal at 40 °C, ramping to 300 °C at 10 °C min^{-1} followed by a 10 min isothermal at 300 °C. The combustion reactor consisted of a quartz tube filled with copper oxide pellets which was maintained at a temperature of 850 °C. Faraday

cups were used to detect ions of *m/z* 44 ($^{12}C^{16}O_2$), 45 ($^{13}C^{16}O_2$ and $^{12}C^{17}O^{16}O$) and 46 ($^{12}C^{18}O^{16}O$). Results were calibrated against reference CO_2 which was injected directly into the source twice at the beginning of the run. All samples were analysed in duplicate and any runs with questionable results were discarded and repeated. External standards were analysed every 4 runs. $\delta^{13}C$ values required correction to take into account the extra carbon atom added during methylation using a mass balance equation [5].

1. Copley M, Berstan R, Dudd S, Docherty G, Mukherjee A, Straker V, Payne S, Evershed R. 2003 Direct chemical evidence for widespread dairying in Prehistoric Britain. *Proc Natl Acad Sci USA* **100**, 1524 – 1529.
2. Correa-Ascencio M, Evershed R. 2014 High throughput screening of organic residues in archaeological potsherds using direct acidified methanol extraction. *Anal Methods* **6,** 1330 – 40.
3. Regert M, Bland H, Dudd S, Bergen P, Evershed R. 1998 Free and bound fatty acid oxidation products in archaeological ceramic vessels. *Proc R Soc B* **265**, 2027 – 2032.
4. Cramp L, Evershed R. 2014 Reconstructing Aquatic Resource Exploitation in Human Prehistory using Lipid Biomarkers and Stable Isotopes. In *Treatise on Geochemistry: Archaeology and Anthropology Vol. 12, 2nd edn* (eds H Holland, K Turekian), pp. 319 – 39. Oxford/Amsterdam, Elsevier.
5. Rieley G. 1994 Derivatization of organic compounds prior to gas chromatographic–combustion–isotope ratio mass spectrometric analysis: identification of isotope fractionation processes. *Analyst* **119**, 915 – 9.

Table S1. Contextual information for pottery discussed. All pottery included is of Neolithic Starčevo-Criş -type

A

Site	Sample_Nr	Bag_Nr	Sherd_nr	Excavation year	Trench/quadrat	Unit	Depth below site datum	Description	Remarks
Vlasac	V-17			2006	Trench 3/2006	32		Cultural layer	
Vlasac	V-29			2006	Trench 3/2006/ quadrant 6 (95/98)	13		Cultural layer	X-find-8

B

Site	Sample_Nr	Bag_Nr	Sherd_nr	Excavation year	Trench/block/quadrat	Spit	Depth below site datum	Description	Remarks
Lepenski Vir	LV-4			1966	Block F	7		Pit	layer 2 within pit going down to house 8
Lepenski Vir	LV-7			1966	Block F	7		Pit	layer 2 within pit going down to house 8
Lepenski Vir	LV-8			1966	Block F	7		Pit	layer 2 within pit going down to house 8
Lepenski Vir	LV-10			1966	Block H I	4		Cultural layer	
Lepenski Vir	LV-13			1967	Quad A V	7		Cultural layer	
Lepenski Vir	LV-30			1968	Quad c II	6		Pit	
Lepenski Vir	LV-32			1968	Quad c II	8		Pit	
Lepenski Vir	LV-35			1968	Quad c II	6		Pit	

C

Site	Sample_Nr	Bag_Nr	Sherd_nr	Excavation year	Trench/block/quadrat	Spit	Depth below site datum	Description
Aria Babi	AB-8R			2004	Test Trench VI/quad. 4	1		Cultural layer
Aria Babi	AB-15			2004	Trench 1	1		Cultural layer
Aria Babi	AB-16			2004	Trench 1	1		Cultural layer
Aria Babi	AB-20			2004	Trench 1	1		Cultural layer
Aria Babi	AB-21			2004	Trench 1	1		Cultural layer

D

Site	Sample_Nr	Bag_Nr	Sherd_nr	Excavation year	Trench/quadrat	Spit	Depth below site datum	Description
Schela Cladovei	SC-01			2008	VII/Q510b	5	0.20-0.25m	Pit
Schela Cladovei	SC-02			2011	VII/Q513a	9	0.40-0.45m	Pit
Schela Cladovei	SC-12			2007	VII	-	Surface horizon	Pit
Schela Cladovei	SC-16			2011	VII/R512d	9	0.40-0.45m	Pit
Schela Cladovei	SC-18			2011	VII/R509d	10	0.45-0.50	Pit
Schela Cladovei	SC-21			2008	VII/T512d	5	0.20-0.25m	Pit
Schela Cladovei	SC-22			2008	VII/T512d	5	0.20-0.25m	Pit
Schela Cladovei	SC-24			2007	VII	-	Surface horizon	Pit
Schela Cladovei	SC-31			2007	VII	-	Surface horizon	Pit
Schela Cladovei	SC-37			2007	VII	-	Surface horizon	Pit
Schela Cladovei	SC-39			2010	VII/R511a	8	0.35-0.40	Pit

E

Site	Sample_Nr	Bag_Nr	Sherd_nr	Excavation year	Trench/profile	Spit	Depth below site datum	Description
Velesnica	VEL-134			1981	-	10		Cultural layer
Velesnica	VEL-136			1981	-	-		Cultural layer
Velesnica	VEL-139			1981	-	-		Cultural layer
Velesnica	VEL-140			1981	-	10		Cultural layer
Velesnica	VEL-141	139		1981	IX	17		Cultural layer
Velesnica	VEL-142	139		1981	IX	17		Cultural layer
Velesnica	VEL-143		1.8A	1981	-	9 (0.5)		Cultural layer
Velesnica	VEL-145	123	82	1981	VIII	9 (0.5)		Cultural layer
Velesnica	VEL-149	139		1981	IX	17		Cultural layer
Velesnica	VEL-150	174	18	1981	VIII	10-16		Cultural layer
Velesnica	VEL-155			1981	-	16		Cultural layer
Velesnica	VEL-157	168	9	1981	VII/7	17		Cultural layer
Velesnica	VEL-159	102	3	1981	VII/7	12		Cultural layer
Velesnica	VEL-164	102		1981	VII7/7	12		Cultural layer
Velesnica	VEL-168	139	109	1981	IX	8		Cultural layer
Velesnica	VEL-170B			1981	-	8		Cultural layer
Velesnica	VEL-170M			1981	-	8		Cultural layer
Velesnica	VEL-179		75	1981	IX	16		Cultural layer
Velesnica	VEL-182	115		1981	VII/7	13		Cultural layer

Table S2. Summary of lipids characterised from the five settlements discussed in the text. LC APAAs = long chain (≥ C_{20} ω-(*o*-alkylphenyl)alkanoic acids; LC DHFAs – long-chain (≥C_{20}) dihydroxy acids. n.d. = not detected

	Sherd location	Vessel features	Lipid conc. (µg g^{-1})	Major lipid biomarkers	Aquatic biomarkers	$\delta^{13}C_{16:0}$ (‰)	$\delta^{13}C_{18:0}$ (‰)	$\Delta^{13}C$ (‰)
Vlasac, Serbia								
V-17	Body		90	Saturated fatty acids (C_{14} – C_{20}) dominated by $C_{16:0}$	LC APAAs	-26.9	-27.8	-0.8
V-29	Body		46	Saturated fatty acids (C_{14} – C_{18}) dominated by $C_{16:0}$; $C_{18:1}$	n.d.	-25.7	-22.9	2.8
Lepenski Vir, Serbia								
LV-4	Rim	Conical bowl	178	Saturated fatty acids (C_{16} – C_{18}) dominated by $C_{18:0}$	n.d. (C_{18} DHFA)	-26.2	-27.9	-1.7
LV-7	Rim	Hole-mouth jar. Red slip	489	Saturated fatty acids (C_{16} – C_{18}) dominated by $C_{16:0}$	LC APAAs, LC DHFAs	-26.0	-26.0	0.0
LV-8	Body	Red slip to interior, finger impressions	831	Saturated fatty acids (C_{16} – C_{20}) dominated by $C_{18:0}$	LC APAAs (C_{18} DHFA)	-25.7	-26.0	-0.3
LV-10	Rim	Conical bowl	1312	Saturated fatty acids (C_{14} – C_{18}) dominated by $C_{18:0}$	LC APAAs	-25.7	-26.0	-0.4
LV-13	Rim	Conical bowl	72	Saturated fatty acids (C_{16} – C_{18}) dominated by $C_{18:0}$	n.d. (C_{18} APAAs only)	-25.0	-27.2	-2.1
LV-30	Rim	Collared jar. Red slip on exterior	356	Saturated fatty acids (C_{16} – C_{18}) dominated by $C_{18:0}$	n.d.	-25.2	-28.1	-2.9
LV-32	Body	Horizontal oblique knob handle	420	Saturated fatty acids (C_{16} – C_{18}) dominated by $C_{18:0}$	LC APAAs	-26.3	-27.3	-1.0
LV-35	Rim	Hole-mouth jar	1298	Saturated fatty acids (C_{16} – C_{18}) dominated by $C_{18:0}$	n.d.	-27.1	-26.2	0.9

Aria Babi, Serbia								
AB-8R	Body		184	Saturated fatty acids (C_{12} – C_{20}) dominated by $C_{16:0}$	LC APAAs	-25.1	-25.0	0.1
AB-15	Body		15	Saturated fatty acids (C_{12} – C_{22}) dominated by $C_{18:0}$	n.d.	-26.7	-27.0	-0.3
AB-16	Body		241	Saturated fatty acids (C_{14} – C_{20}) dominated by $C_{16:0}$	LC APAAs	-31.0	-31.0	0.0
AB-20	Body		27	Saturated fatty acids (C_{12} – C_{22}) dominated by $C_{18:0}$	n.d.	-25.8	-26.6	-0.8
AB-21	Body		88	Saturated fatty acids (C_{12} – C_{20}) dominated by $C_{18:0}$	n.d.	-25.9	-26.6	-0.6

Schela Cladovei, Romania								
SCL-01	Rim	Conical bowl	3672	Saturated fatty acids (C_{14} – C_{20}) dominated by $C_{16:0}$	LC APAAs	-26.1	-26.1	0.0
SCL-02	Rim	Hole mouth jar	834	Saturated fatty acids (C_{14} – C_{20}) dominated by $C_{16:0}$	LC APAAs	-28.4	-28.0	0.4
SCL-12	Rim	Plate	241	Saturated fatty acids (C_{16} – C_{18}) dominated by $C_{18:0}$	n.d.	-25.9	-26.6	-0.7
SCL-16	Rim	Necked bowl	562	Saturated fatty acids (C_{14} – C_{18}) dominated by $C_{18:0}$	n.d.	-26.1	-30.5	-4.4
SCL-18	Rim	Hole-mouth jar	68	Saturated fatty acids (C_{16} – C_{18}) dominated by $C_{18:0}$	LC APAAs	-25.2	-26.0	-0.8
SCL-21	Rim	Conical bowl	703	Saturated fatty acids (C_{14} – C_{20}) dominated by $C_{16:0}$	LC APAAs (C_{18} DYFA)	-28.6	-28.5	0.1
SCL-22	Rim	Conical bowl	376	Saturated fatty acids (C_{16} – C_{22}) dominated by $C_{16:0}$	LC APAAs	-28.0	-27.9	0.1
SCL-24	Rim	Hole-mouth jar	123	Saturated fatty acids (C_{16} – C_{18}) dominated by $C_{18:0}$	n.d.	-25.9	-26.0	-0.2
SCL-31	Rim	Conical bowl	161	Saturated fatty acids (C_{16} – C_{18}) dominated by $C_{18:0}$	n.d.	-26.5	-27.1	-0.7
SCL-37	Rim	Conical bowl	812	Saturated fatty acids (C_{14} – C_{20}) dominated by $C_{18:0}$	n.d.	-26.0	-26.5	-0.4
SCL-39	Body		495	Saturated fatty acids (C_{16} – C_{18}) dominated by $C_{18:0}$	n.d.	-24.3	-25.5	-1.2

Velesnica, Serbia								
VEL-134	Body		23	Saturated fatty acids (C_{14} – C_{20}) dominated by $C_{16:0}$	LC APAAs; LC DHFAs	-27.3	-27.4	-0.1
VEL-136	Rim	Handle; incised rim	33	Saturated fatty acids (C_{14} – C_{20}) dominated by $C_{16:0}$	LC APAAs; LC DHFAs	-27.2	-27.7	-0.5
VEL-139	Body	Decorative lines	13	Saturated fatty acids (C_{14} – C_{20}) dominated by $C_{16:0}$	LC APAAs; LC DHFAs	-26.6	-26.6	0.1
VEL-140	Rim	Very thick, undulating rim	139	Saturated fatty acids (C_{14} – C_{20}) dominated by $C_{16:0}$	LC APAAs; LC DHFAs	-26.9	-26.6	0.3
VEL-141	Rim	Large diameter. Pinching to rim	26	Saturated fatty acids (C_{14} – C_{20}) dominated by $C_{16:0}$	n.d.	-25.7	-27.1	-1.4
VEL-142	Rim	Large diameter	11	Saturated fatty acids (C_{14} – C_{20}) dominated by $C_{16:0}$	LC APAAs; LC DHFAs	-26.6	-26.0	0.5
VEL-143	Rim/body/ base	Incised rim	12	Saturated fatty acids (C_{14} – C_{20}) dominated by $C_{16:0}$	n.d.	-24.9	-26.4	-1.5
VEL-145	Rim	Finger impressions to rim	69		n.d.	-26.4	-26.8	-0.4
VEL-149	Rim	Large diameter	142	Saturated fatty acids (C_{14} – C_{20}) dominated by $C_{16:0}$ and $C_{18:0;}$ TAGs (C_{46} – C_{54})	n.d.	-25.6	-26.2	-0.6
VEL-150	Rim		43	Saturated fatty acids (C_{14} – C_{20}) dominated by $C_{16:0}$, ketones (C_{31} – C_{35})	LC APAAs; LC DHFAs	-27.8	-27.9	-0.1
VEL-155	Rim		20	Saturated fatty acids (C_{14} – C_{20}) dominated by $C_{16:0}$	LC APAAs; LC DHFAs	-27.1	-26.1	1.0
VEL-157	Rim		26	Saturated fatty acids (C_{14} – C_{20}) dominated by $C_{16:0}$, ketones (C_{31} – C_{35})	LC APAAs; LC DHFAs	-27.6	-27.4	0.2
VEL-159	Rim	Large diameter. Finger impressions to rim	25	Saturated fatty acids (C_{14} – C_{20}) dominated by $C_{16:0}$	LC APAAs; LC DHFAs	-26.3	-26.1	0.2
VEL-164	Base/lower body		20	Saturated fatty acids (C_{14} – C_{20}) dominated by $C_{16:0}$	n.d.	-26.4	-27.6	-1.2
VEL-168	Mid body	Knobbles	12	Saturated fatty acids (C_{14} – C_{18}) dominated by $C_{16:0}$, ketones (C_{31} – C_{35})	n.d. (C_{18} APAAs only)	-25.9	-25.1	0.8

VEL-170B	Base	Knobbles in half horseshoe	77	Saturated fatty acids (C_{14} – C_{20}) dominated by $C_{16:0}$	n.d.	-26.3	-26.9	-0.6
VEL-170M	Rim	Knobbles in half horseshoe	84	Saturated fatty acids (C_{14} – C_{20}) dominated by $C_{16:0}$	n.d.	-25.3	-24.9	0.4
VEL-179	Mid body Type I		226	Saturated fatty acids (C_{14} – C_{20}) dominated by $C_{18:0}$, traces TAGs C_{48} – C_{54})	n.d.	-27.9	-29.3	-1.4
VEL-182	Base/lower body		73	Saturated fatty acids (C_{14} – C_{20}) dominated by $C_{16:0}$	LC APAAs; LC DHFAs	-26.5	-25.7	0.8

Table S3. Summary of published faunal information from Vlasac 1970-71 excavations [1, 2] and 2006-9 excavations [2], Lepenski Vir [3] and Schela Cladovei [4, with revisions]. Quantification is shown by NISP.

Site	Vlasac				Lepenski Vir						Schela Cladovei			
	1970 – 71 Mesolithic		2006 – 2009 Late Mesolithic		LV I		LV II		LV III		Mesolithic		Neolithic	
	Spec.	%	Spec.	%	Spec.	%	Spec.	%	Spec.	%	Spec.	%	Spec.	%
**Mammals**														
Cattle									375	15.83			424	18.26
Aurochs	54	0.16	1	0.00	14	3.3	7	3.41	174	7.34	75	4.46	53	2.28
Bos sp.			1	0.00										
Sheep/goat									81	3.42			402	17.31
Sheep													53	2.28
Chamois	22	0.06							2	0.08				
Red deer	6732	19.59	199	0.57	115	27.12	111	54.15	862	36.39	157	9.33	230	9.91
Red deer antler (if specified)											77	4.58	66	2.84
Roe deer	510	1.48	48	0.14	4	0.94	1	0.49	36	1.52	33	1.96	41	1.77
Roe deer antler (if specified)											2	0.12		
Fallow deer			1	0.00										
Domestic pig									8	0.34			200	8.61
Wild pig	1175	3.42	77	0.22	10	2.36	6	2.93	211	8.91	48	2.85	39	1.68
Sus sp.			6	0.02										
Wild ass									7	0.3				

Wild cat	45	0.13	4	0.01					1	0.04	2	0.12		
Lynx	5	0.01	1	0.00					6	0.25				
Marten	248	0.72	8	0.02	6	1.42	3	1.46	3	0.13				
Badger	58	0.17	1	0.00	3	0.71			7	0.3	2	0.12	1	0.04
Brown bear	169	0.49	6	0.02					27	1.14			1	0.04
Dog	1914	5.57	326	0.94	21	4.95	23	11.22	140	5.91	21	1.25	29	1.25
Wolf	103	0.30	4	0.01					7	0.3	3	0.18	1	0.04
Fox	30	0.09	7	0.02					1	0.04			1	0.04
Canis sp.			6	0.02										
Small carnivore	13	0.04												
Beaver	71	0.21	6	0.02	2	0.47			4	0.17				
Squirrel	5	0.01												
Hare	22	0.00	4	0.01					7	0.3	6	0.36	14	0.6
Hedgehog	1	0.00	2	0.01										
European mole			1	0.00										
Mammals unid.	?		10675	30.77										
**Amphibians and reptiles**														
Greek tortoise											64	3.8	28	1.21
Pond tortoise	317	0.92	40	0.12										
Frog/toad											2	0.12	5	0.22

**Birds**	146	0.42	38	0.11	6	1.42	1	0.49	10	0.42				
**Fish**														
Ascipenseridae sp.	19	0.06	7	0.02							265	15.75	102	4.39
Beluga	21	0.06												
Sterlet											50	2.97	29	1.25
Catfish	2283	6.64	85	0.25	3	0.71	5	2.44	22	0.93	46	2.73	30	1.29
Cyprinids	5230	15.22	6566	18.93	86	20.28	1	0.49	14	0.59				
Pike	11	0.04	1	0.00							13	0.77	19	0.82
Pike-perch											2	0.12	4	0.17
Carp	1552	4.52									466	27.69	303	13.05
Bream											18	1.07	14	0.6
Orfe											2	0.12	1	0.04
Small cyprinids	5230	15.22									329	19.55	232	9.99
Other fish	8372	24.37	16570	47.76	154	36.32	47	22.93	364	15.37				
**Total**	**34358**		**34691**		**424**		**205**		**2369**		**1683**		**2322**	

1. Bökönyi S. 1978 The vertebrate fauna of Vlasac. In *Vlasac: Mezolitsko naselje u Djerdapu, vol. 2* (ed M Garašanin), pp. 35 – 65. Belgrade, Srpska akademija nauka i umetnosti.
2. Borić D, French C, Stefanović S, Dimitrijević V, Cristiani E, Gurova M, Antonović D, Allué E, Filipović D. 2014 Late Mesolithic lifeways and deathways at Vlasac (Serbia). *Journal of Field Archaeology* **39**, 4 – 31.
3. Bökönyi S. 1970 Animal remains from Lepenski Vir. *Science* **167(3926)**, 1702 – 4.

4. Bartosiewicz L, Boroneanţ V, Bonsall C, Stallibrass S. 2001 New data on the prehistoric fauna of the Iron Gates: a case study from Schela Cladovei, Romania. In *From the Mesolithic to the Neolithic: proceedings of the International Archaeological conference held in the Damjanich Museum of Szolnok, September 22–27, 1996* (eds R Kertész, J Makkay), pp. 15 – 21 (Budapest, Archaeolingua).

Figure S1. Partial gas chromatograms showing typical fatty acid distributions from A) Velesnica, Serbia (analysed as trimethylsilyl esters), B) Lepenski Vir, Serbia (analysed as fatty acid methyl esters) and C) from Schela Cladovei, Romania (analysed as fatty acid methyl esters). Labels - *X*:*Y* – free fatty acid with *X* carbon atoms and degree of unsaturation *Y*. D) mass chromatograms from a Velesnica residue run in full scan (extracting *m/z* 105) and SIM mode (*m/z* 105 and $M^{+\bullet}$290 and *m/z* 105 and $M^{+\bullet}$ 318), demonstrating the enhanced sensitivity of the latter for the identification of APAAs.

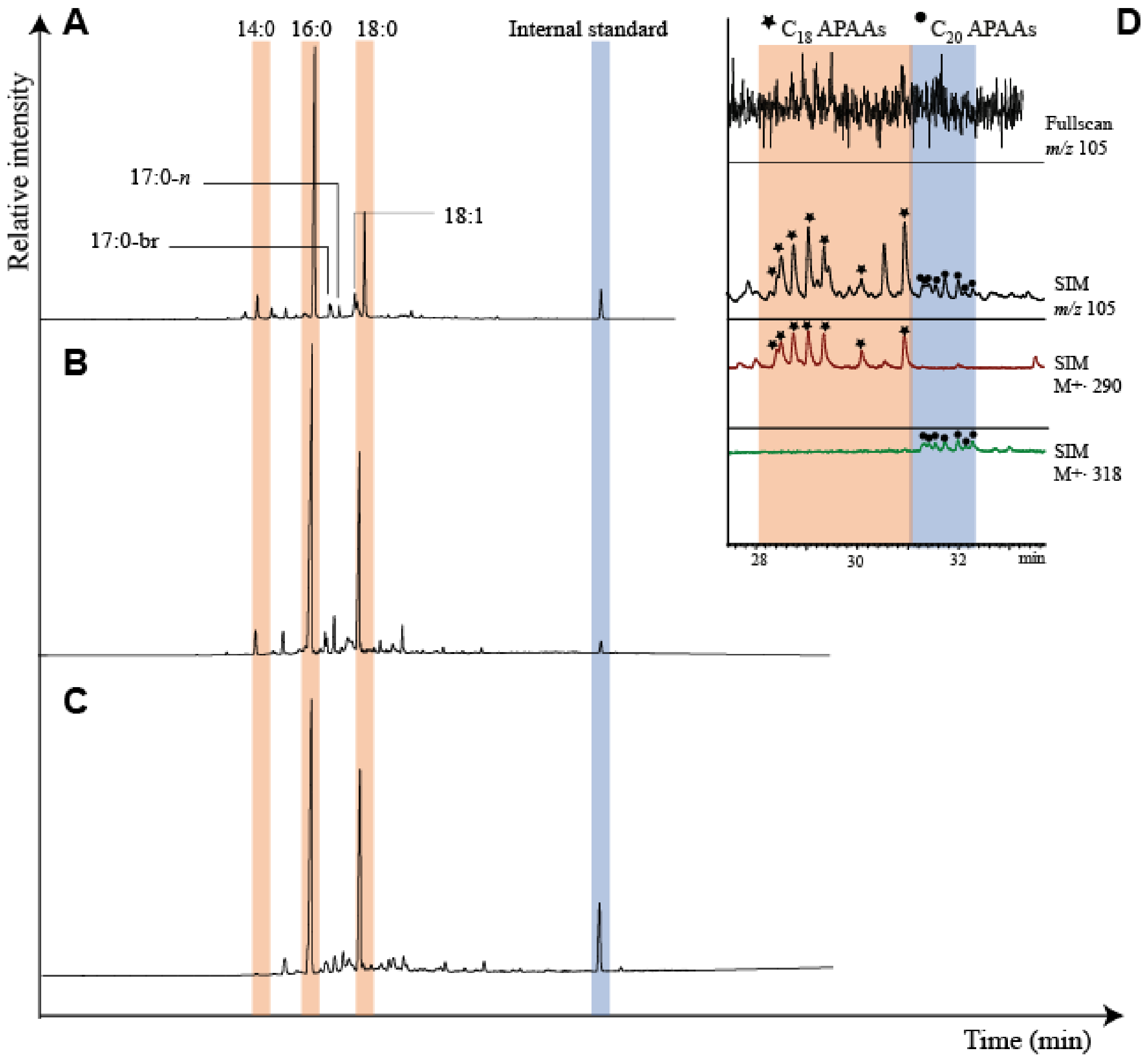

Figure S2. Plot of $\delta^{13}C_{16:0}$ against $\Delta^{13}C$ ($\delta^{13}C_{18:0} - \delta^{13}C_{16:0}$) mean and full ranges from analyses of reference species [1-3], with hypothetical mixing lines between mean values for groups. The bi-coloured squares on the mixing lines show where the expected value from (large) a 50:50 mix of fats and (small) a 75:25 mix of non-ruminant: ruminant fat would lie, based upon average $\delta^{13}C$ values and fatty acid proportions from reference fats [1-3], demonstrating the strong bias against aquatic fats isotopically when mixed with ruminant fats.

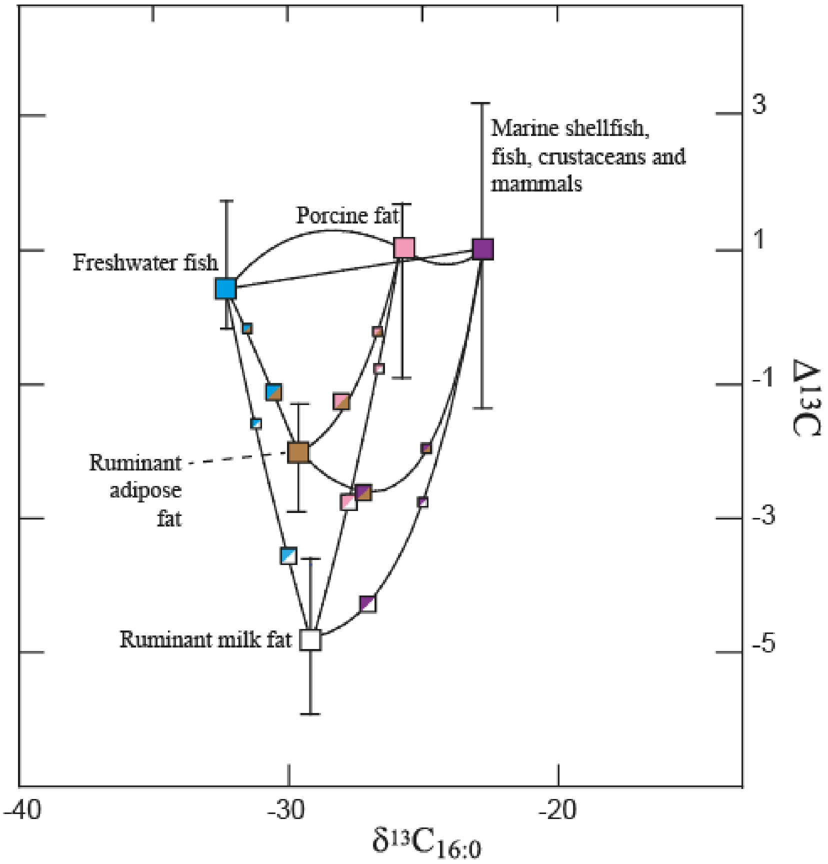

1. Copley M, Berstan R, Dudd S, Docherty G, Mukherjee A, Straker V, Payne S, Evershed R. (2003 Direct chemical evidence for widespread dairying in Prehistoric Britain. *Proc Natl Acad Sci USA* **100**, 1524 – 1529.
2. Cramp L, Evershed R. 2014 Reconstructing Aquatic Resource Exploitation in Human Prehistory using Lipid Biomarkers and Stable Isotopes. In *Treatise on Geochemistry: Archaeology and Anthropology Vol. 12, 2nd edn* (eds H Holland, K Turekian), pp. 319 – 39. Oxford/Amsterdam, Elsevier.
3. Stear N. 2008 *Changing patterns of animal exploitation in the prehistoric Eurasian Steppe: an integrated molecular, stable isotope and archaeological approach*. Ph.D. thesis, University of Bristol, UK.
